# Juana la Loca

**DOI:** 10.1017/S1478951526101990

**Published:** 2026-02-25

**Authors:** William Breitbart

**Affiliations:** Department of Psychiatry & Behavioral Sciences, Memorial Sloan Kettering Cancer Center, New York, NY, USA

I am immersed in insanity
A woman
Of passion and beauty.
A woman of dark eyes
and wild hair.
The taste of Madrid
is still in my mouth.
The jamon, the tortilla,
The Reina Sofia museum,
La Latina.
I am in love with a woman
Not Dora Maar,
but with the same eyes
We stare at each other
across the table.
The tapas plates are piled high.
She is insane to love me.
I am a jealous man.
I cannot leave her.
Juana la Loca.

